# Mass Reduction Techniques for Short Backfire Antennas: Additive Manufacturing and Structural Perforations

**DOI:** 10.3390/s23218765

**Published:** 2023-10-27

**Authors:** Yewande Mariam Aragbaiye, Dustin Isleifson

**Affiliations:** 1 Department of Electrical & Computer Engineering, University of Manitoba, Winnipeg, MB R3T 5V6, Canada; 2 Centre for Earth Observation Science, Clayton H. Riddell Faculty of Environment, Earth and Resources, University of Manitoba, Winnipeg, MB R3T 2N2, Canada

**Keywords:** 3D printing, additive manufacturing, FDM, metal plating, perforation technique, short backfire antenna, weight reduction

## Abstract

This paper presents novel approaches for reducing the mass of the classical short backfire (SBF) antenna by using additive manufacturing and structural perforations. We first investigated techniques to create a 3D-printed structure with a conductive coating material. This approach resulted in a significant mass reduction (70%) compared with the conventional metallic structure. We performed parametric simulation studies to investigate the effects of the manufacturing process and showed that there was practically no difference in the performance. The largest source of error was the surface roughness and the conductivity of the metal paint. In a second design, we created perforations in the structure to further reduce the mass. We performed parametric studies to optimize mass reduction and to characterize the effects of the perforations and the surface roughness introduced during the 3D-printing process on the antenna. Antenna prototypes were fabricated and tested. The masses of the perforated 3D printed antenna were approximately 30% and 20% of the original aluminum design, respectively (70% and 80% reductions in mass, respectively). The good agreement among the original design, simulation, and measurements demonstrated the effectiveness of the approach.

## 1. Introduction

The classic short backfire (SBF) antenna comprises two reflectors, one bigger and one smaller, spaced about 0.5λ_0_, with a dipole feed between them and a rim enclosing the larger reflector [1]. In cases where conventional antennas, such as single end-fire antennas or paraboloids, are too large or challenging to build for medium-gain applications, the SBF antenna, which generates a gain of about 13 to 20 dBi, can be utilized. In hazardous conditions such as space, maritime, and Arctic conditions, it has been used for remote sensing due to its durability, compact structure, and moderate gain [2]. Drones have recently been equipped with antennas and radiating systems, and these devices are used for anything from remote sensing to search and rescue missions. Such antennas must be small and efficient and have a low mass and low profile to reduce battery consumption and increase flight times. The SBF antenna is an excellent option because it can be designed and manufactured to satisfy these requirements. While several SBF antennas that have been designed satisfy most of these requirements, many still fall short regarding the low mass requirement.

Traditional SBF, reflector, and horn antennas can range from 500 g [3] to 8 kg [4] or even higher in weight, depending on the type and size of the antenna. In remote sensing applications, where antennas are installed on drones with weight restrictions, or in space applications, where the entire system weight significantly influences launch costs, heavy-weight antennas can pose a challenge. A system performs better and costs less to operate when the antenna weight is reduced. Numerous techniques have reportedly been employed to considerably minimize antenna mass. These include 3D-printing techniques and perforation methods [5,6,7], which can also be applied to the SBF antenna.

The first approach to reducing the SBF antenna’s mass involved fabricating the antenna using additive manufacturing techniques, also known as 3D printing. Over the last few years, antenna and electromagnetic constructions have been printed using various 3D-printing methods, including photonic polymerization, stereo lithography, micro stereo lithography, Polyjet printing, and fused deposition modeling (FDM). Each technique provides advantages and disadvantages that are best suited to producing various types of antennas. For instance, the FDM technique, which extrudes filament materials from a heated nozzle, is typically the most affordable 3D-printing process while also offering the advantages of a large selection of filaments and the ability to print with many materials at once. Laser or UV light is used in the stereolithography (SLA) and polymerization technique to cure resin components into the desired 3D shape. Therefore, they can attain a resolution significantly higher than that of FDM, making them suitable for producing smaller structures. Because the materials used in these printers are essentially a light-curable resin, they cannot be altered to the same degree as FDM filaments [8].

For most metallic antennas, such as the SBF antenna, a conductive surface is usually required to interface with the input port. Poor conductors, which are suitable for 3D printed electrical circuits, are not appropriate for antenna applications since the losses negatively influence radiation efficiency. To fabricate metallic antennas, specialist metal 3D printers that can directly print metallic materials such as aluminum, steel, titanium, and cobalt through direct metal laser sintering (DMLS) provide a good option [9,10]. An alternative and more affordable method for producing metallic antennas involves using regular filaments or resins during 3D printing and then coating the pieces with conductive materials [11,12]. Jet metal processing (JMT), aerosol spray coating with copper and nickel conductive paints, and brush painting with silver conductive paint are some metallization techniques that have been used for similar applications [11]. The latter 3D-printing method offers several advantages over pure metal antennas, including being lighter in weight and incorporating complex and finely detailed 3D designs associated with additive manufacturing techniques. A significant area for improvement in 3D-printed antennas is the surface roughness introduced due to the layer-by-layer printing method. High surface roughness degrades the antenna’s performance and can lead to a significant gain loss; this becomes a bigger issue at submillimeter wavelengths. A post-processing step of polishing the surface is required before the metal coating is applied to minimize the surface roughness issues.

The perforation technique is another method used for the weight reduction of reflector and horn antennas. This technique has been achieved in the literature by either creating holes/slots in the antenna [13,14,15,16] or using a mesh-like, grid-like, or textile material with several tiny holes to build the antenna [17,18]. In [13], the metal-sheet perforation technique was used in the design of a waveguide-fed horn to achieve a lightweight feature; two-thirds of the weight was reduced while the electrical and mechanical capabilities of the antenna’s structure were maintained. A lightweight mesh metal material was used in [18,19] instead of solid metal to design reflector antennas and significantly reduce their mass. In contrast, the antenna presented in [17] achieved 50% weight reduction by fabricating the left side of the hyperbolic sub-reflector with grid wires and composite technology. Although this technique effectively reduces the weight of the antenna, it is almost impossible to avoid some losses due to the removed portions of the antenna and the redistribution of the surface current. Choosing the right hole/slot size and the spacing distance between them is essential for minimizing these losses [14].

The work presented in this paper aims to reduce the weight of SBF antennas while meeting the operation specifications of a C-band remote sensing system. This is done by implementing an improved aluminum SBF antenna prototype using the 3D-printing technique in the first candidate design. The perforation technique was applied to the second antenna to further reduce the overall weight. A simulation study was completed to characterize the performance, and a prototype was fabricated using a 3D printer to verify the concepts.

Section 2 presents the design concepts and the simulation results of both antenna designs. Simulations were performed using ANSYS Electronics Desktop 2021 R2©. Section 3 explains the fabrication process and measurement results used to verify the proposed concept. Section 4 gives a conclusion and recommendations for the next steps in the design flow.

## 2. Design Concept

**Figure 1:**
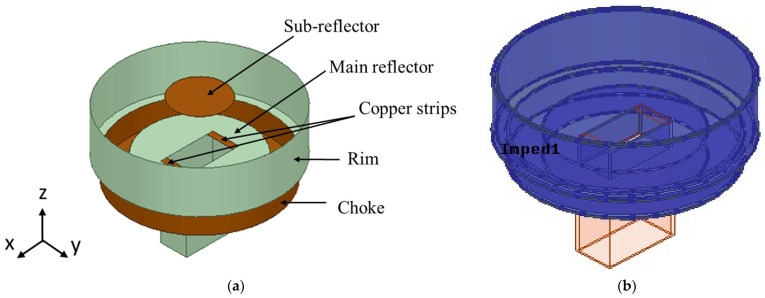
Modified SBF antenna: (**a**) geometry; (**b**) impedance boundary (blue region).

The proposed SBF antenna designs are based on the improved SBF antenna presented in [3] and shown in Figure 1a. It is a modified waveguide-fed SBF antenna with a choke. The purpose of the choke is to improve the gain performance of the antenna by changing the surface current distribution. The WR159 waveguide feed (with dimensions a = 40.386 mm and b = 20.193 mm) was used for all of the designs in this manuscript. It was selected because of its high gain, power-handling capabilities, and ease of fabrication.

Table 1 summarizes the antenna design specifications. These performance metrics were developed using design specifications from current C-band remote sensing systems and by comparing them to other designs that are already published in the literature. The SBF antennas in this study were designed to target the C-band (4–7 GHz) with a center operating frequency of 5.5 GHz, which corresponds to a wavelength of λ_0_ = 54.54 mm in free space. The antenna is expected to have a minimum impedance bandwidth of 500 MHz and a minimum gain of 14 dBi within the frequency band of operation to perform efficiently. In this study, the antennas presented were designed to weigh less than 50% of the aluminum prototype.

### 2.1. 3D-Printed SBF Antenna

Based on previous research on conventional reflector antennas, we looked for ways to reduce the mass of the SBF antenna by using lighter materials, such as thermoplastics, for the design process. Designing with thermoplastic cannot be easily done when using conventional milling fabrication and often requires a 3D-printing fabrication process. Polylactic acid (PLA) and acrylonitrile butadiene styrene (ABS) were used as the 3D printer filaments for building the antenna prototypes presented in this manuscript because of their low cost and strong mechanical characteristics. The metal coating was applied using the aerosol spray technique with the MG Chemicals Super Shield nickel conductive coating and the MG Chemicals Super Shield Silver Conductive Paint. These methods and materials were chosen because of their cost-effective and fast application process. They have also been reported to produce excellent results [20].

Because 3D printing is a layer-by-layer process, surface roughness is introduced and can be detrimental to the performance of high-frequency antennas. Consequently, it is crucial to simulate the effects of different surface roughness values on the performance of the SBF antenna. The body of the antenna was modeled in HFSS using a material with a relative permittivity of 2.8 and a loss tangent of 0.005 to roughly approximate the properties of the ABS plastic for the simulation of the 3D-printed SBF antenna. [21]. To simulate the behavior of the metal spray paint on the thermoplastic, an impedance boundary condition was applied to the surface of the ABS material [22]. This was done by first selecting all of the faces associated with the ABS material and then assigning the selected faces to an impedance boundary, as shown in Figure 1b (the blue region). After applying this boundary, the resistance value in Ω/sq could be entered to approximate the surface resistance of the material. The metal layer’s thickness and level of surface roughness both affected the impedance value (Ω/sq) of this surface boundary.

**Figure 2:**
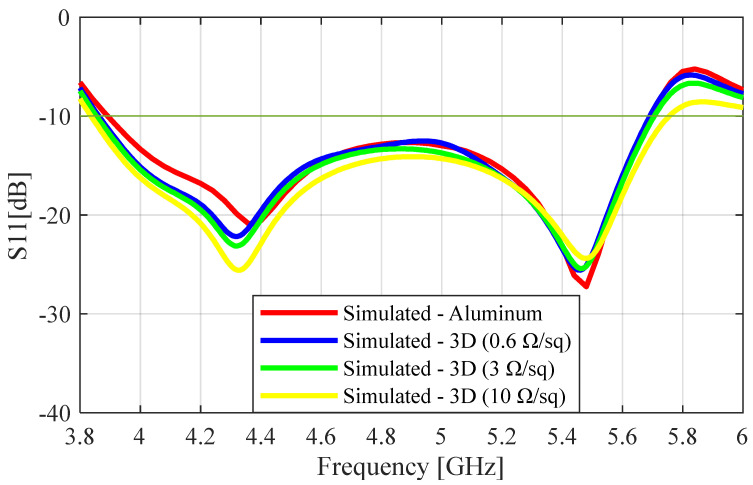
Simulated S11 plots for the aluminum and 3D-printed prototypes.

Surface impedance increases with surface roughness while decreasing with the metal layer’s conductivity and thickness [23]. Given that the coating was manually applied, it was anticipated that the thickness of the metal layer would vary somewhat across the surface of the ABS material. We investigated the effect of the variation in thickness on the performance of the 3D-printed SBF antenna by gradually reducing the diameter of the main reflector from 120 mm to 119 mm with a step decrease of 0.1 mm. We found that the variation in thickness did not significantly affect the antenna’s performance, i.e., the gain was constant within ±0.1 dB. Several surface impedances were used to examine the behavior of the antenna due to the rough surface that was anticipated from the fabrication process. Additionally, we contrasted the outcomes of two scenarios: one in which the antenna was entirely coated and another in which only its interior walls were coated. The simulated plots were obtained for surface resistance values of 0.6 Ω/sq, 3 Ω/sq, and 10 Ω/sq. The surface resistance for the Super Shield Nickel Conductive Coating was 0.6 Ω/sq, so we took this value as the minimum surface resistance that we could achieve experimentally without additional surface roughness from the 3D-printing process. We included the simulation results for 3 Ω/sq because it gave a gain loss of about 0.6 dB between the simulation (with foam) and measured result, which was the same gain loss that was obtained for the aluminum prototype when the simulated and measurement gain results were compared. We also provided the results for 10 Ω/sq to show the effects of higher surface resistance on the antenna’s performance. Figure 2 shows the simulated S_11_ results of the aluminum and the 3D-printed prototype for SR values of 0.6 Ω/sq, 3 Ω/sq, and 10 Ω/sq. From this figure, we can see that the impedance bandwidth of the SBF antenna increased with the surface resistance. The 10 dB impedance bandwidth of the aluminum was calculated to be 1.8 GHz (37.5%), while those of the 3D-printed antenna were 1.84 GHz (38.5%), 1.86 GHz (39%), and 1.92 GHz (40%) for SR values of 0.6 Ω/sq, 3 Ω/sq, and 10 Ω/sq, respectively. This increase in impedance bandwidth with increasing roughness is supported by the equations given in [24], showing that impedance bandwidth is directly proportional to surface roughness.

**Figure 3:**
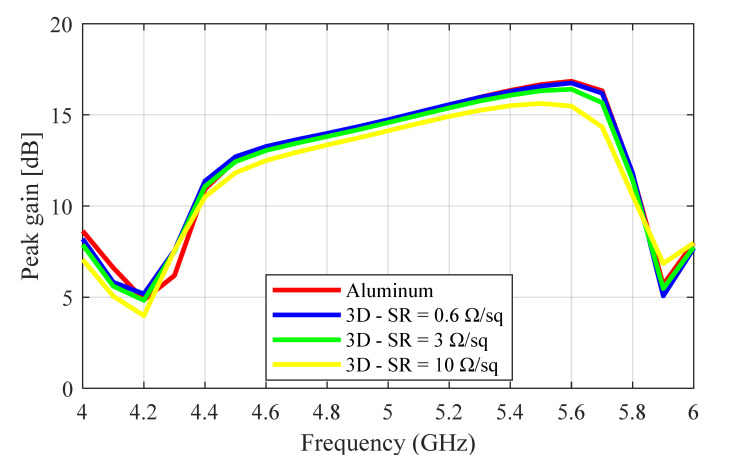
Simulated realized gain plots (ϕ = 0°) comparing the aluminum prototype and the 3D-printed prototypes with surface roughness (SR) values of 3 Ω/sq and 10 Ω/sq.

The peak realized gain results were calculated for several surface impedance values. The summary of the results is given in Table 2, where we can see that the realized gain decreased slowly as the surface impedance increased. The performance of the entirely coated antenna was slightly better than that when only the inner walls were coated. Figure 3 shows the realized gain results comparing the aluminum prototype and the 3D-printed protypes with surface roughness (SR) values of 0.6 Ω/sq, 3 Ω/sq, and 10 Ω/sq. As expected from the previous result, we can see that the gain decreased with the increase in the SR value. The aluminum prototype had the highest peak gain of 16.7 dBi at 5.5 GHz, followed by the 3D-printed prototype with SR values of 0.6 Ω/sq, 3 Ω/sq, and 10 with peak gain values of 16.5 dBi, 16.1 dBi, and 15.6 dBi, respectively. The gain bandwidth of operation that satisfied the 14 dBi requirement extended from 4.8 to 5.75 GHz (950 MHz) for the aluminum antenna and from 4.8 to 5.75 GHz (950 MHz), 4.9 to 5.8 GHz (900 MHz), and 5.0 to 5.5 GHz (500 MHz) for the 0.6 Ω/sq, 3 Ω/sq, and 10 Ω/sq 3D-printed prototypes, respectively. This proved that reducing the surface roughness increased the peak gain and the gain bandwidth. At high frequencies, the surface roughness values on conductive materials can be in a similar range to the wavelength. When this happens, surface irregularities and defects alter the conductor’s electrical characteristics, particularly affecting the metal’s effective conductivity. Equations (1)–(4) presented in [24] show the relationships between effective conductivity, 3 dB bandwidth, and surface roughness. From the equations, we can see that increasing the surface roughness of the antenna’s conductive surface leads to a decrease in its effective conductivity and quality factor, which, in turn, increases the 3 dB bandwidth. As a result of the decreased conductivity, there is an increase in the signal loss (gain loss) and dispersion, which is one of the prominent effects of surface roughness [24].

**Figure 4:**
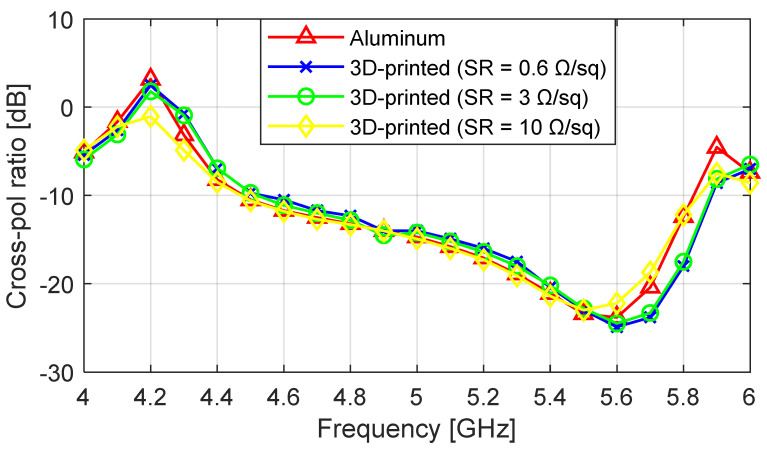
Simulated worst-case cross-polarization ratio at ϕ = 45°; comparison of the aluminum prototype and the 3D-printed prototypes with surface roughness (SR) values of 3 Ω/sq and 10 Ω/sq.

The simulated worst-case cross-polarization ratio curves for the aluminum and 3D-printed antenna designs are given in Figure 4. The minimum cross-polarization ratio for the aluminum was −24 dB, and the 0.6 Ω/sq, 3 Ω/sq, and 10 Ω/sq 3D-printed prototypes gave minimum cross-polarization ratio values of −24.5 dB, −24 dB, and −22 dB, respectively, at 5.6 GHz. Due to the very close agreement between the plots of the aluminum and the 3D-printed antennas with different SR values, it can be concluded that increasing the surface roughness of the antenna had a very small effect on the cross-polarization ratio.

In this section, the effects of surface roughness and resistance on the performance of the 3D-printed SBF antenna are studied and compared with the performance of the aluminum SBF antenna. We noticed that the increased surface roughness and resistance introduced by the 3D-printing and metallization processes did not have a significant effect on the reflection coefficient (S_11_); on the other hand, the gain decreased as the surface roughness and resistance increased. It was, therefore, important to keep it as low as possible by polishing the antenna before metallization and using a highly conductive metal spray. Comparing the weight of the ABS plastic (1.0 g/cm^3^) to that of the aluminum metal (2.7 g/cm^3^), the greatest advantage of the 3D-printing fabrication process compared to the conventional method is a 70% reduction in mass.

### 2.2. Perforated SBF Antenna

The perforation technique is a relatively simple concept that involves purposefully creating a series of sub-wavelength slots in the antenna, meaning that the slots are significantly smaller than the antenna’s wavelength (λ_0_ = 54.54 mm) in order to achieve weight reduction. Because the diameter of the holes is less than λ_0_, the transmitted EM wave does not radiate from these holes; hence, they do not significantly affect the performance of the antenna. Numerical analysis of the unperforated SBF antenna was implemented using HFSS (ANSYS Electronics Desktop 2021 R2©) in the previous section. An interesting phenomenon observed during the analysis was that most of the current density was concentrated in the main reflector, while the rim had a much lower current density. By taking advantage of this current distribution, portions of the rim were removed without disturbing the performance.

**Figure 5:**
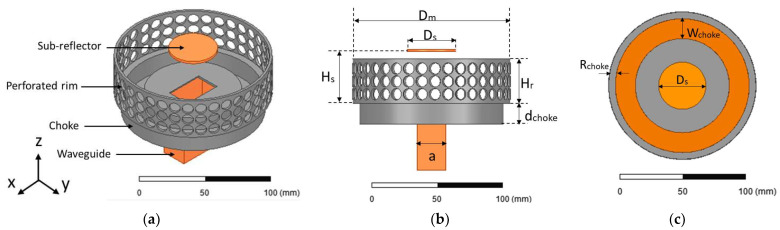
Perforated SBF antenna: (**a**) isometric view; (**b**) front view; (**c**) top view.

Our goal was to increase the size of the holes as much as possible to maximize the mass reduction while maintaining the same performance. After several studies on different shapes, sizes, and positioning of the slots on the antenna’s rim based on this technique, we propose the waveguide-fed perforated SBF antenna shown in Figure 5. To simulate this design in HFSS, the perforated rim of the antenna was first modeled as a rectangular sheet with a length and width equal to the circumference of the large reflector and the height of the solid rim, respectively. For the perforations, 37 × 3 circular sheets were modeled on the rectangular sheet in a periodic manner. The subtract function was used to remove the circles from the rectangular sheet, which was then wrapped and thickened to form the shape of the rim.

We carried out several parametric simulation studies to understand the effects of the perforations on the antenna performance and determine a design that satisfied the requirements. Different perforation radii (0–5 mm) were simulated to investigate their effects on the performance of the antenna, such as those on the gain, impedance bandwidth, and cross-polarization ratio.

**Figure 6:**
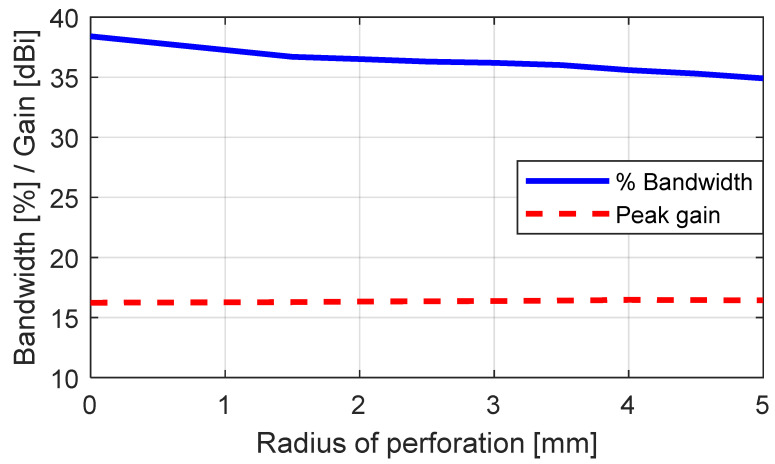
The variations in the peak gain and impedance bandwidth as a function of the radius of perforation.

**Figure 7:**
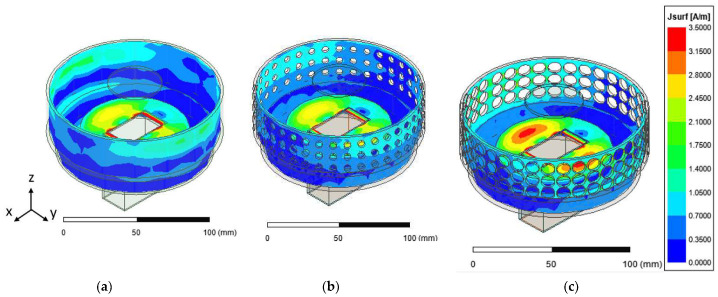
Surface current density for (**a**) the unperforated SBF antenna, (**b**) the perforated SBF antenna (PR = 2.5 mm), and (**c**) the perforated SBF antenna (PR = 4.5 mm).

A plot of the peak gain and the impedance bandwidth as a function of the radius of perforation at an operating frequency of 5.5 GHz is given in Figure 6. Increasing the perforation radius (PR) did not have a significant effect on the peak gain (~16 dBi), while the impedance bandwidth could be seen to gradually decrease (38.4–34.9%). Figure 7 shows the surface current distribution of the unperforated and perforated SBF antennas (PR = 2.5 mm and PR = 4.5 mm); it could be observed that perforating the rim of the antenna altered the surface current distribution depending on the radius of perforation. The perforated antenna with a PR of 4.5 mm had a higher surface current density close to the waveguide feed and on the rim compared to the antenna design with a PR of 2.5 mm due to the reduced surface area. This also contributed to the changes noticed in the bandwidth and gain results.

**Figure 8:**
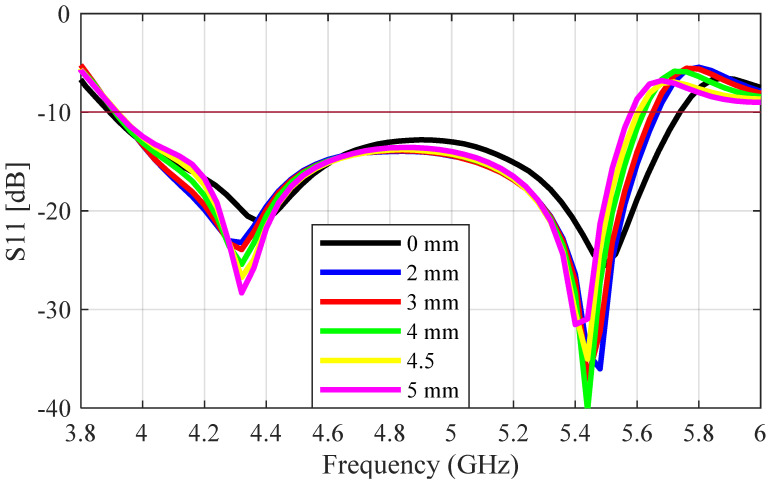
Simulated S_11_ plot for the 3D-printed perforated antennas with perforation radii of 0 mm, 2 mm, 3 mm, 4 mm, and 5 mm.

**Figure 9:**
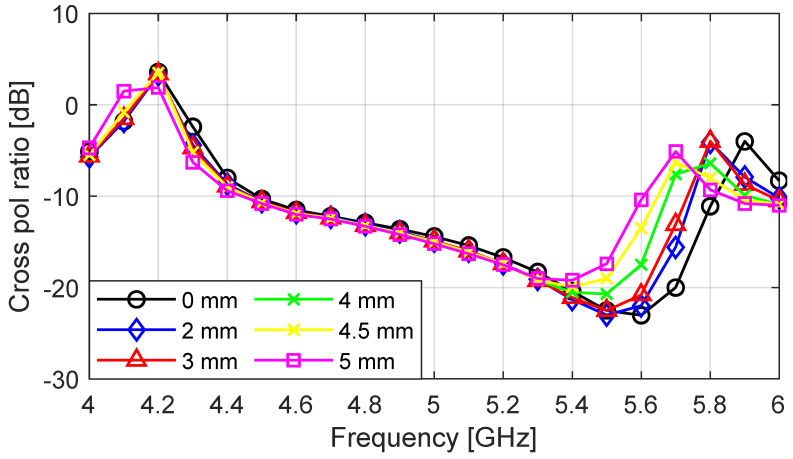
Simulated cross-polarization ratio for perforation radii from 0 mm to 5 mm at ϕ = 45°.

The S_11_ result for the perforated antenna (radius = 1 mm to 5 mm) is compared with that of the unperforated antenna in Figure 8; the plots are in very close agreement, with a very small decrease in the bandwidth as the perforation radius increases. The impedance bandwidth values decreased from 38.4% to 34.9 as the radius of perforation increased from 0 mm to 5 mm. The cross-polarization ratio of the perforated antenna as a function of frequency is given in Figure 9 for a perforation radius increasing from 0 mm to 5 mm. The cross-polarization ratio did not change between 4 and 5.3 GHz as the perforation radius increased. As the frequency increased from 5.3 to 6 GHz, the unperforated antenna (PR = 0 mm) provided the lowest value of −24 dB at 5.6 GHz. The cross-polarization ratio started to increase as the radius of perforation increased, with PR = 5 mm having a minimum value of −20 dB at 5.4 GHz. The perforated antenna with PR = 4.5 mm was chosen as the optimum design, as it provided the highest percentage of mass reduction while maintaining the other performance metrics of the unperforated antenna.

Simulation studies on the perforated SBF antenna showed that increasing the size of the perforations minimally decreased the impedance bandwidth. This was because of the surface current redistribution and impedance change around the waveguide aperture caused by the discontinuities introduced by the perforations. The peak gain at 5.5 GHz stayed almost the same for increasing perforation size, which is what was expected for perforation sizes that were significantly smaller than one wavelength. The gain of the antenna did not significantly change because the current distributions at the upper rim and aperture were not substantially affected. Consequently, the radiation pattern was not drastically changed either. As a result of the higher current density associated with this design, it was important to reduce the surface resistance caused by the 3D-printing and metallization processes to avoid extreme gain loss. Compared to the unperforated prototype, the removal of portions of the antenna’s rim in addition to the use of lightweight plastics for fabrication resulted in an 80% overall reduction in the antenna’s mass.

**Figure 10:**
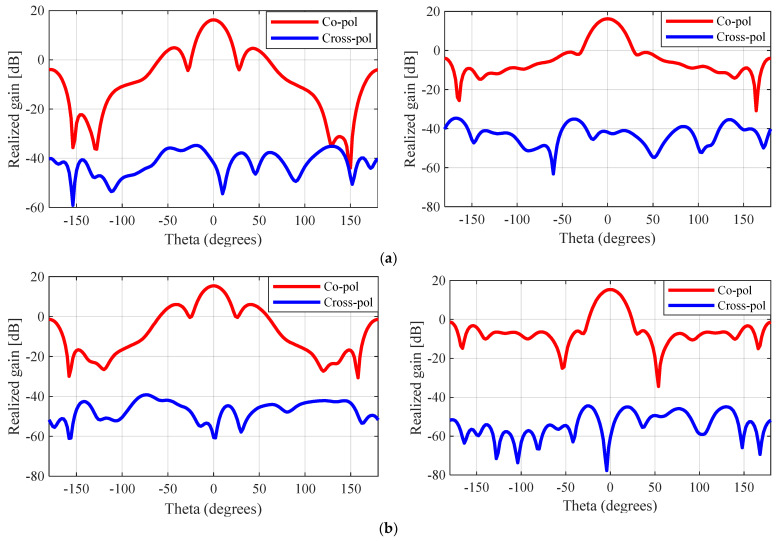

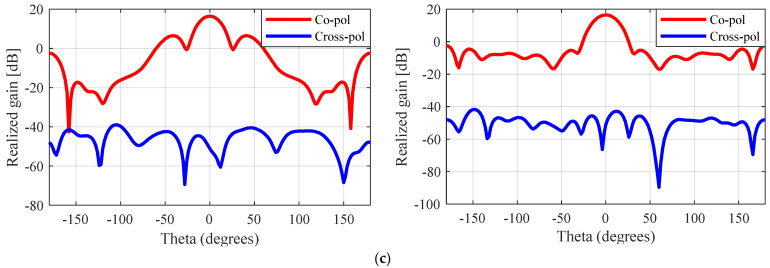
Simulated E-plane (ϕ = 0°) (**left side**) and H-plane (ϕ = 90°) (**right side**) radiation patterns for the 3D-printed SBF antenna at 5.5 GHz: (**a**) unperforated SBF antenna; (**b**) nickel-coated perforated SBF antenna; (**c**) silver-coated perforated SBF antenna.

The simulated E-plane and H-plane radiation patterns (θ = −180–180°) for the unperforated, nickel-coated, and silver-coated perforated SBF antennas are given in Figure 10. It can be observed from these plots that the behavior of the antenna changed slightly after the perforation technique was applied. The front-to-back ratio for the unperforated antenna was 20.1 dB (16.1 dBi − (−4 dBi)), while that of the perforated antenna was reduced to 17.0 dB (15.6 dBi − (−1.4 dBi)) and 18.9 dB (16.2 dBi − (−2.7 dBi)) for the nickel- and silver-coated prototypes, respectively. This change in behavior can be attributed to the surface current and impedance changes that were caused by applying the perforations to the antenna and the surface roughness.

## 3. Fabrication and Testing

The Airwolf Axiom 3D printer was used to fabricate the 3D-printed prototypes at the University of Manitoba’s machine shop. The 3D-printing technique used fused deposition modeling (FDM). To make sure that the antenna was operating at optimum performance, the first post-processing step involved polishing the surface of the 3D-printed antenna by sanding it. Since the plastic was not conductive, the last step involved metallizing the model using a conductive metal spray paint. The entire antenna surface was covered by several layers of paint to achieve a good conductive and reflective surface.

**Figure 11:**
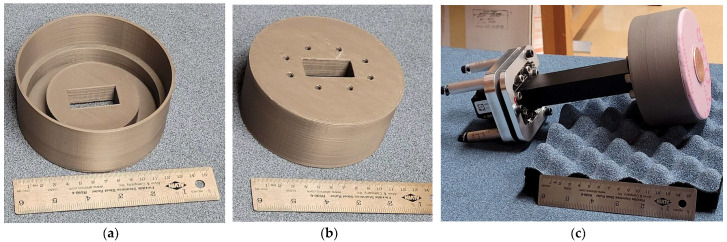
Fabricated 3D-printed antenna: (**a**) top view; (**b**) bottom views; (**c**) fully assembled with the waveguide feed attached.

The fabricated solid 3D-printed antenna is shown in Figure 11; ABS plastic (with a density of 1.04 g/cm^3^) was used as the thermoplastic filament. After the antenna was printed, it was sprayed by hand using the MG Chemicals Super Shield Nickel Conductive Coating. The thickness of the metal coating was measured to be approximately 0.3 mm, which was much thicker than the skin depth of nickel at 5.5 GHz (0.07246 μm). A piece of foam material (30 mm thick) with an approximate dielectric constant of 1.05 was placed inside the main reflector cavity to position the sub-reflector at the required height. Figure 11c shows the fully assembled antenna with the WR-159 waveguide attached to the main reflector of the SBF antenna. In this section, it is important to note that all of the simulated results provided in this section took the effects of the foam material into consideration.

**Figure 12:**
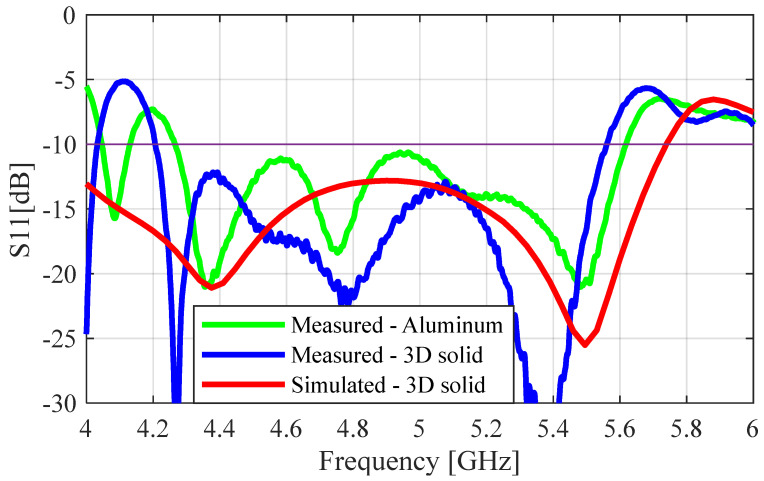
Simulated and measured S_11_ plots for the solid 3D-printed antenna compared to the aluminum prototype.

The measured dimensions for both the unperforated and perforated 3D printed antennas have been provided in Table 3. The S-parameters (S_11_) of the 3D-printed antennas were measured using a Keysight PNA Network Analyzer (N5224B), and the measured far-field results were obtained in the Compact Antenna Test Range of the University of Manitoba. The S_11_ performance measurement of the waveguide adaptor (WR-159) was found to have complex impedances for frequencies below 5.2 GHz, which resulted in multiple oscillations that resulted in characteristic differences between the simulation and measurements (documented in [3]). To improve the S_11_ results of the SBF-2 antenna (aluminum), two copper tape strips were adhered to the aperture of the waveguide. This improved the impedance match by creating an inductive iris, and this approach was also applied to the 3D-printed antenna to obtain a similar result. The measured S_11_ plots for the aluminum SBF antenna and the simulated and measured S_11_ results for the solid 3D-printed prototype are provided in Figure 12. Comparing the results shown in Figure 12, the interference from the waveguide can be seen in the measured results of both the aluminum and 3D-printed prototypes. There was a close agreement between the S_11_ results of the aluminum and the solid 3D-printed SBF antennas; the measured impedance bandwidths were 1.35 GHz (27.3%) and 1.38 GHz (27.7%), respectively.

**Figure 13:**
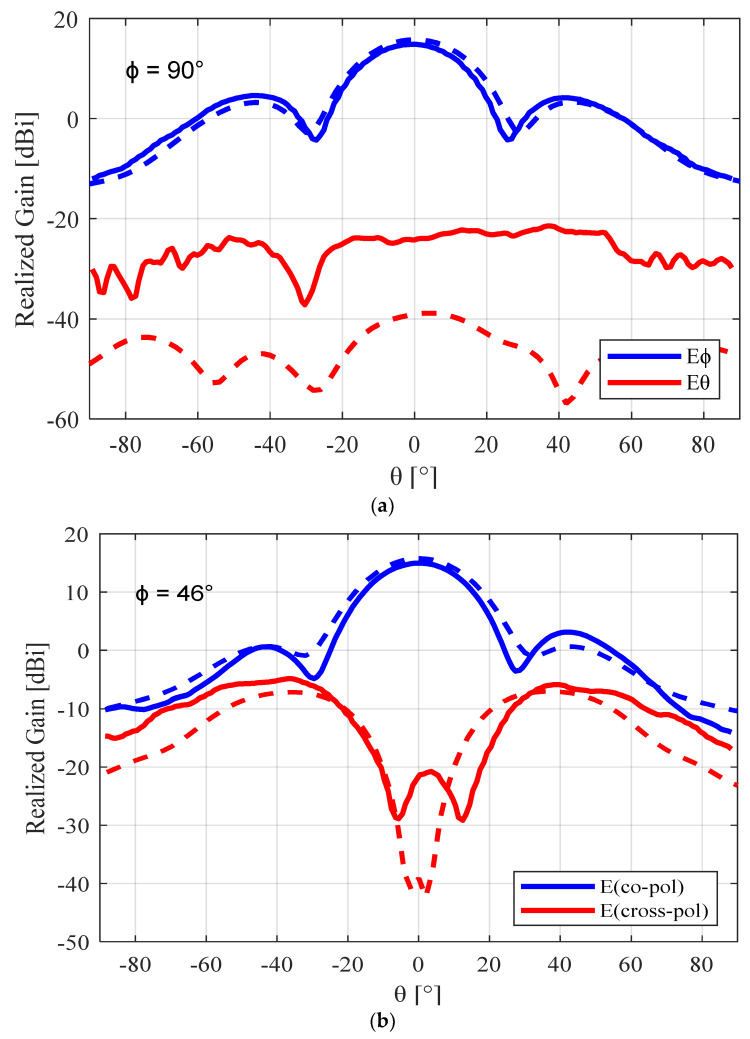

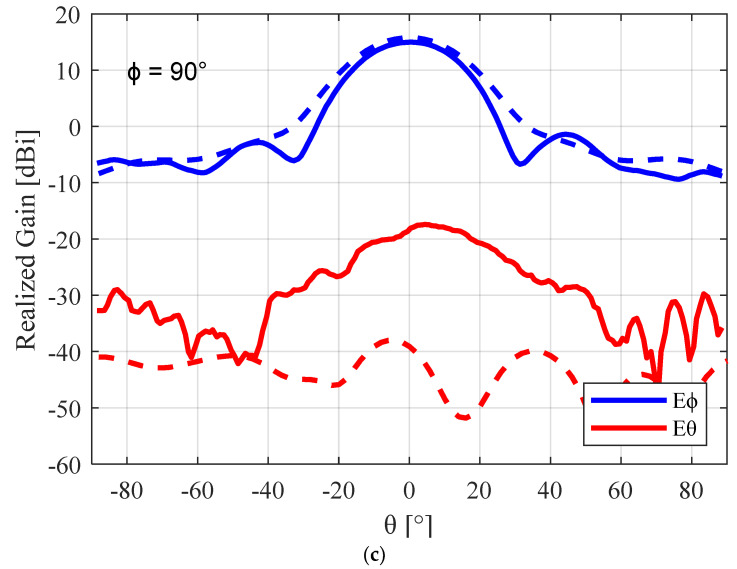
Radiation pattern results for the 3D-printed SBF antenna at (**a**) ϕ = 0°, (**b**) ϕ = 46°, and (**c**) ϕ = 90°. The dashed lines represent the simulated results, while the solid lines represent the measured results.

The simulated and measured radiation pattern results for the 3D-printed SBF antenna are shown in Figure 13. The measured peak gain was 15 dBi, whereas the simulated peak gain was 15.63 dBi (including the effects of the foam insert and using a surface impedance of 3 Ω/sq). If we consider the 0.4 dB loss from the insufficient bonding between the waveguide flange and the bottom of the primary reflector, the remaining 0.23 dB loss can be attributed to the insertion loss from the waveguide to coaxial cable connector. We obtained the measured cross-polarization ratio at 5.5 GHz from Figure 13b using Ludwig’s third definition [6]. The peak value was at ϕ = 46° and θ = 38°, giving a worst-case cross-polarization ratio of −19.9 dB. This was very close to the measured worst-case cross-polarization ratio of the aluminum prototype, which was −20.9 dB.

Comparing the measured peak gain for the 3D-printed SBF antenna (15 dBi) to that of the aluminum prototype, which was measured to be 15.7 dB [3], we noticed a gain loss of about 0.7 dB. The major cause of this gain loss can be attributed to some degree of surface roughness present on the surface of the 3D-printed antenna due to the layer-by-layer printing technique (the layers can be seen in Figure 11 for a visual inspection; the surface of the printed antenna also did not feel as perfectly smooth as the aluminum metal surface). Another reason for the gain loss can be because of the imperfect metallization process. Equivalent losses like these have been documented in the literature for horn antennas constructed using other 3D printing + metallization techniques [25,26,27]. However, it is hoped that better metal deposition, such as additional electroplating methods, will be able to reduce the loss.

**Figure 14:**
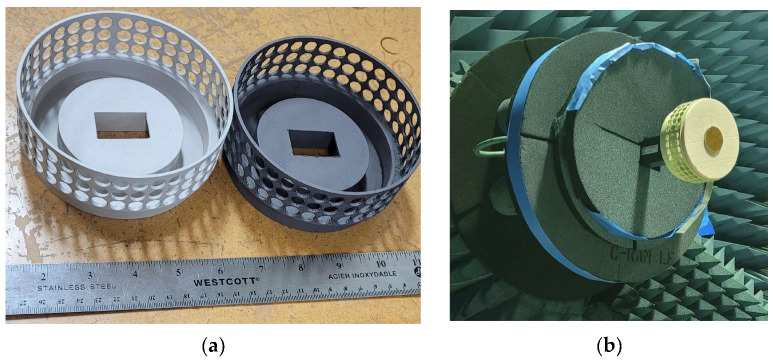
Fabricated perforated 3D-printed SBF antennas: (**a**) silver-coated (**Left**) and nickel-coated (**Right**); (**b**) in the compact antenna testing range for radiation patterns.

For the fabrication of the perforated SBF antenna, a perforation radius of 4.5 mm was used. The PLA thermoplastic filament was the preferred material for the perforated antenna, as printing with ABS plastic resulted in many deformities and much stringing around the perforated areas. Stringing occurs when tiny plastic strings are left on a 3D-printed model. This happens mostly when the extruder temperature is too high, since it can cause the filament to overheat and ooze out of the nozzle. In the case of an ABS filament that has a higher melting point of 200 °C, this can be particularly problematic. To reduce the stringing problem, PLA with a lower melting point of 173 °C was used, and it produced a better finish. We also decided to compare the performance of two different metal paints: silver (Ag) and nickel (Ni) conductive paints. To compare the performance of the nickel conductive paint with the silver conductive paint with a lower resistivity (1.2 × 10^−6^ Ω·m) and superior EMI/RFI shielding, two perforated SBF antenna prototypes were 3D printed. The fabricated perforated 3D-printed SBF antennas are shown in Figure 14a; the prototype on the left is silver-coated, while the one on the right side is nickel-coated. The iris that was added in the previous design using copper tape strips was included in this design and was 3D printed along with the rest of the antenna piece. The far-field measurement setup of the perforated SBF antenna in the compact range is shown in Figure 14b. It was found experimentally and through simulations that the same thickness of foam that was used for the previous unperforated antenna was not suitable for the perforated SBF antenna, as it significantly degraded the gain. Therefore, a thinner foam with a thickness of 10 mm was used to support the sub-reflector instead of the 30-mm-thick foam that was used for the previous design.

**Figure 15:**
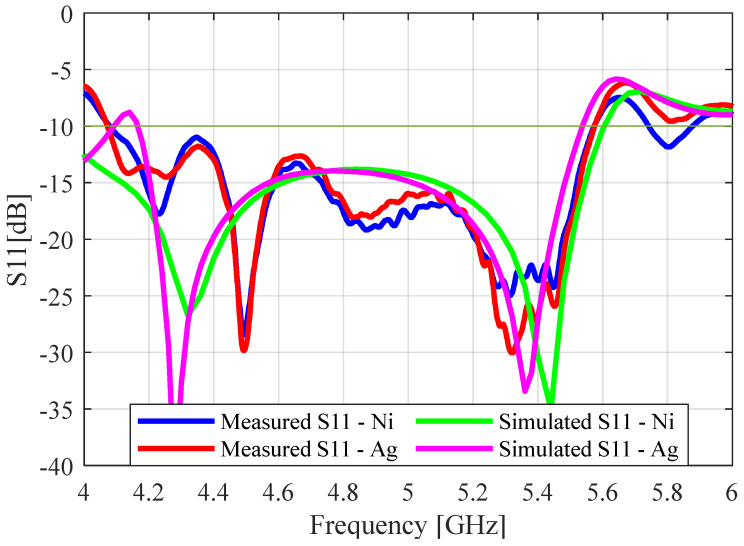
Simulated and measured S_11_ plots for the perforated 3D-printed antenna.

The two antenna prototypes were connected to the waveguide feed and measured using the network analyzer. The measured and simulated (S_11_) results for both the nickel- and silver-coated protypes are given in Figure 15. As before, the effects of the waveguide adaptor can be seen in the measured results when comparing them with the simulated results. The simulated S_11_ values for the nickel and silver prototypes were obtained using SR values of 1 Ω/sq and 0.1 Ω/sq, respectively. The measured S_11_ values of the nickel-coated and silver antennas were in very close agreement, as they had the same impedance bandwidth of 1.52 GHz (31.5%). This showed that using the silver paint instead of the nickel paint did not significantly affect the reflection coefficient of the perforated SBF antenna.

**Figure 16:**
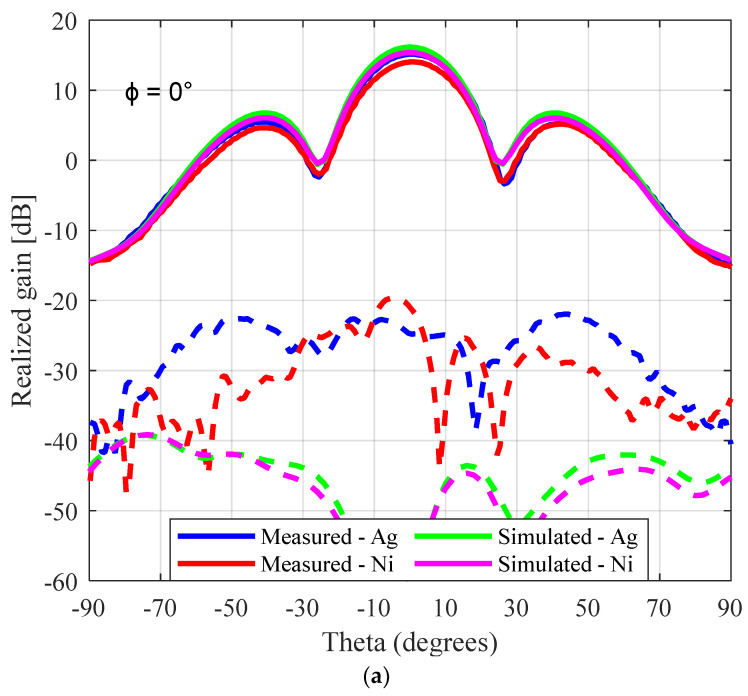

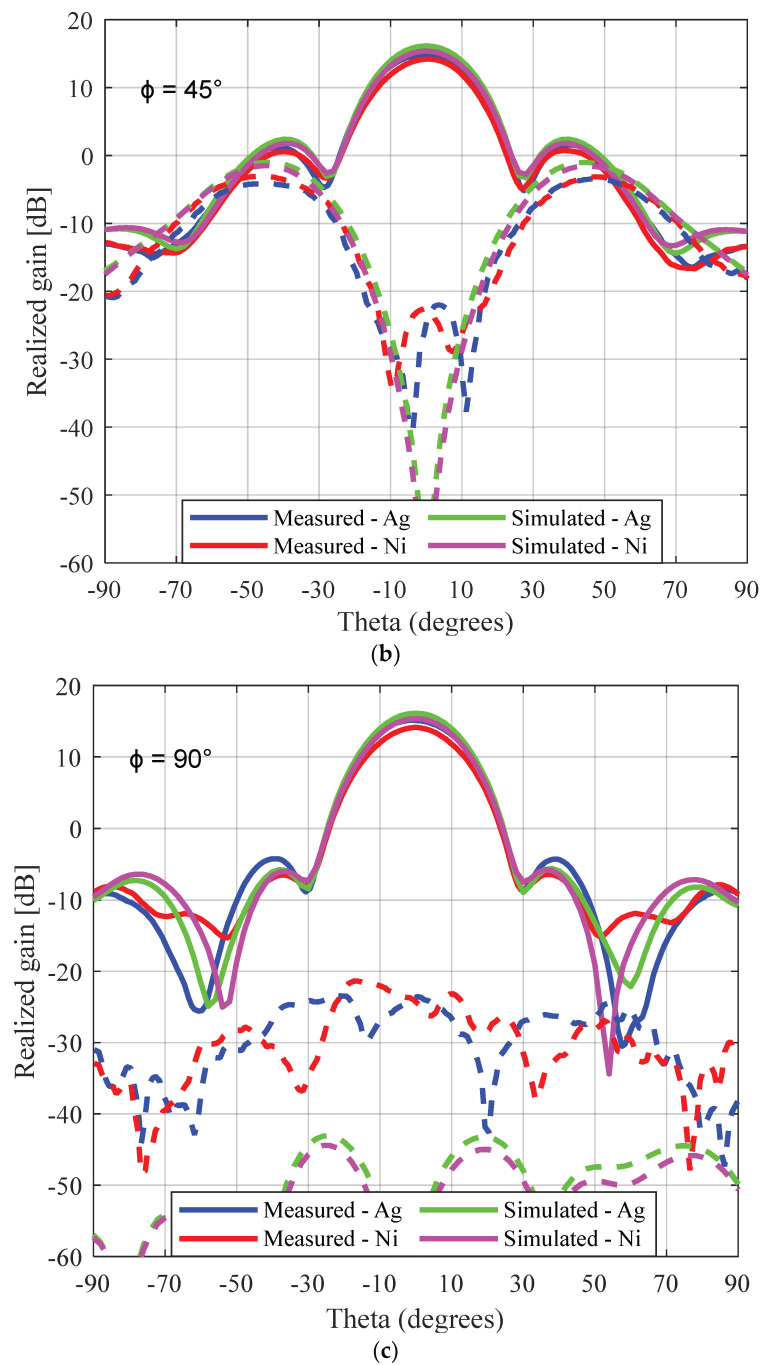
Radiation pattern results for the perforated 3D-printed SBF antenna at (**a**) ϕ = 0°, (**b**) ϕ = 45°, and (**c**) ϕ = 90°. The dashed lines represent Eθ, while the solid lines represent Eϕ.

Figure 16 gives the measured and simulated radiation pattern results for both the nickel- and silver-coated perforated SBF antenna prototypes. The measured peak gain for the nickel- and silver-coated antennas were 14.2 dBi and 15.2 dBi, respectively, while the simulated gain was 15.3 dBi for nickel (SR = 1 Ω/sq) and 16.2 dBi for silver (SR = 0.1 Ω/sq). It is obvious from these results that using the silver paint increased both the simulated and measured gain significantly by 0.9 dB and 1.1 dB, respectively, compared to the nickel paint. As we did in the previous section, assuming about ~0.6 dB loss was due to the imperfect coaxial cable to waveguide connection and the primary reflector not being perfectly bonded to the waveguide flange; the remaining ~0.4 dB loss can be attributed to the imperfections introduced by the 3D-printing process.

Through simulation, the surface of the perforated rim was found to have a higher surface current compared to the unperforated prototypes, making the losses higher for the perforated antenna; therefore, it was important to use the silver paint with a higher conductivity to reduce these losses. The measured worst-case cross-polarization ratios at 5.5 GHz were calculated to be −18.7 dB and −17.3 dB for the silver-coated and nickel-coated antennas, respectively, at ϕ = 45° and θ = −45.

The performance of the aluminum, unperforated, and perforated 3D-printed prototypes is compared in Table 4. Although some gain loss was experienced, employing the 3D-printing fabrication technique alone reduced the weight of the antenna from 462 g to 139 g (70% reduction), while incorporating the perforation technique led to an overall 80% reduction (462 g to 103 g). In comparison with the original aluminum structure, the 3D-printed antenna’s performance was acceptable and complied with the design requirements.

To highlight the benefits of our innovative antenna, we compared it with existing low-mass antenna designs, as shown in Table 5. It can be seen that the antenna presented in [13] employed the metal-direct-printing 3D-printing technique using metallic filaments, which can be heavier in weight than plastic filaments. Therefore, even after applying the perforation technique, the reduction in mass was only about 63.8% compared to the 80% that was achieved in this study by using plastic filaments. We also observed that applying the perforation technique alone, as presented in [15,17], resulted in a reduction in the mass of the antenna of about 50%. Antennas that made use of thermoplastic filaments [28,29] had similar mass reduction percentages to those of the antennas presented in this work. Although the K-band Horn antenna [29] achieved an 80% weight reduction, the gain loss due to the 3D-printing process was almost 1 dB, while the gain loss for our perforated antenna was 0.5 dB, with a much lower cost of fabrication. Compared to these works that achieved similar weight reduction results, an optimization study can be carried out on the current perforated antenna design to further reduce the antenna mass to less than 20%—for example, by optimally packing the circular holes in a lattice-type structure and investigating combinations of multiple perforation sizes.

## 4. Conclusions

Design procedures for decreasing the mass of an SBF antenna were presented in this manuscript. The proposed antenna designs have several appealing characteristics, including low cross-polarization, high gain, and good impedance bandwidth, while being lower in mass than existing SBF antennas with comparable attributes. To achieve mass reduction for the first design, an SBF antenna was implemented using additive manufacturing technology, which is also known as 3D printing. The process involved using a 3D printer to fabricate an antenna prototype with thermoplastic material and then metallizing the surface using a conductive paint. While this technique is very beneficial for achieving low-mass designs, the 3D-printing process introduces some level of surface roughness, which can cause performance degradation. It is, therefore, important to address this problem by polishing the antenna to reduce the effect of the surface roughness. The first antenna design (unperforated design) was fabricated. The simulated and measured results of the 3D-printed antenna were in close agreement with the results of the aluminum prototype for both S11 and radiation patterns. There was a 0.7 dB drop in the measured gain for the 3D-printed antenna compared to the aluminum prototype. This gain loss can be attributed to surface roughness and the lower conductivity of the metal coating. Employing the additive manufacturing technique in the design of the SBF antenna led to a significant reduction in the weight of approximately 70%.

To further reduce the mass of the antenna, holes were introduced into the 3D-printed SBF antenna structure, resulting in a perforated SBF antenna design. From the simulation studies, we observed that the performance of the antenna did not change significantly with perforations that were much smaller than a wavelength. Two prototypes of this design were fabricated and coated using silver and nickel metal paints to compare their performances. The antennas were tested, and all performance metrics were achieved at 5.5 GHz. The silver paint was found to increase the gain of the perforated SBF antenna from 14.1 dBi (gain obtained for the nickel coating) to 15.2 dBi (1.1 dB increase in gain). While the silver paint increased the gain of the antenna, the financial cost was about 10 times the cost of the nickel paint for the same quantity. Therefore, the nickel paint was more cost effective than the silver paint. According to the results obtained, using both the perforation and 3D-printing techniques reduced the mass of the antenna by 80% while still meeting the design requirements, with a minimal gain loss of 0.5 dB (compared to the aluminum SBF antenna).

In future work, we aim to address the surface roughness issues by using LiDAR to measure and evaluate the precise surface roughness values of the 3D-printed surface and investigate the use of chemicals such as acetone for polishing the antenna. We also plan to conduct more research on advanced metallization techniques such as electroplating and jet metal processing. By reducing the surface roughness introduced by the 3D-printing process and obtaining uniform and precise metal deposition, we hope to further improve the gain of the 3D-printed antenna and get the performance as close as possible to that of the aluminum prototype. We also plan to carry out more optimization studies on the perforated antenna design, such as optimally packing the circular holes in a lattice-type structure and investigating combinations of multiple perforation sizes.

## Figures and Tables

**Table 1:** Design specifications for the 3D-printed SBF antennas.

Specifications	Metric
Operating Frequency	5.5 GHz
Mass	<50% of the original aluminum construction
Realized Gain	>14 dBi
Impedance Bandwidth	500 MHz

**Table 2:** The effect of surface impedance on the realized gain of the 3D-printed antenna [7].

Surface Impedance (Ω/sq)	Gain (dBi) Entire Body Coated	Gain (dBi) Inner Walls Coated
0.6	16.45	16.25
1	16.36	16.2
3	16.05	16.0
5	15.91	15.8
7	15.78	15.65
10	15.58	15.4

**Table 3:** Measured dimensions of the 3D-printed antenna prototypes.

Parameter	Description	Value (mm)
Dm	Diameter of the main reflector	119.7
Ds	Diameter of the sub-reflector	38.2
Hr	Height of the rim	32.7
Hs	Height of the sub-reflector	38.5
Rchoke	Distance between the outer diameter of the choke and the rim	5.5
dchoke	Depth of the choke	16.1
Wchoke	Width of the choke	16.1

**Table 4:** Comparison between the performance of the aluminum and 3D-printed prototypes.

	Aluminum SBF Antenna	3D-Printed SBF Antenna	Perforated 3D SBF Antenna (Silver Coated)	Perforated 3D SBF Antenna (Nickel Coated)
Weight (g)	462.5	139	103	103
Gain (dBi)	15.7	15.0	15.2	14.2
Impedance Bandwidth (%)	27.3%	27.7%	31.5	31.5
Front–back ratio (dB)	20.7	20.1	18.7	17.0

**Table 5:** Performance comparison of the proposed antennas with other antenna designs.

	Description	Mass Reduction Technique	Operating Frequency Range (GHz)	Impedance BW (%)	Peak Gain (dBi)	Mass Reduction (%)
This work	3D-printed SBF antenna	3D printing and nickel plating	4–7	27.7	15.0	70
This work	Perforated 3D-printed SBF antenna	3D printing and perforation technique	4–7	31.5	15.2	80
[13]	Lightweight Perforated Horn Antenna	3D printing (metal-direct-printing technology) and perforation technique	8–12	40	11.2	63.8
[15]	Lightweight dual cylindrical reflector antenna	Perforation technique	9–14	17	3	50
[17]	Compact Low-Weight High-Gain Broadband Cassegrain Antenna	Grid wires and composite technology	8–10	50	34.5	50
[28]	Horn Antenna Covered with a 3D-Printed Meta-surface	3D printing and copper plating on ABS printed horn	10–18	66.67	25	80
[29]	K-band Horn Antenna with Charge-Programmed Deposition 3D Printing	Charge-Programmed Deposition 3D Printing	17.5–20.5	15.8	14.31	80

## Data Availability

Data are available within the article.
